# Supplementary material for: Transcriptomic and macroevolutionary evidence for phenotypic uncoupling between frog life history phases
Source: Nat Commun. 2017 May 15;8:15213. doi: 10.1038/ncomms15213 (PMC5440664; doi:10.1038/ncomms15213)
Supplement: Supplementary Information — Supplementary Figures, Supplementary Tables, Supplementary Methods and Supplementary Refernces [file ncomms15213-s1.pdf]

Supplementary Methods

Phase-specific gene expression: additional information on *Xenopus* gene expression data. Xenbase.org entries used for comparison of phase-specific gene expression came from a variety of sources with the most common being publications (24,425 entries), large-scale screens (16,233 entries), and community-submitted entries (6,480 entries). Most entries that were obtained from community submission, large scale screens and publications originated from immunohistochemistry and in situ hybridization experiments, whereas other sources provided cDNA Library/EST data. Most entries (50,992) were obtained from whole organism. Five curation methods were included: automated curation with nucleotide BLAST (120,381 entries), none (21,835 entries), stages manually curated (15,547 entries), complete manual curation (7,947 entries), and incomplete manual curation (1,254 entries).

Analysis of the mantellid radiation: taxonomic and phylogenetic framework. The frog subfamily Mantellidae at present contains 212 species[1] but a large number of additional confirmed candidate species have been identified[2,3]. These are genetically strongly divergent lineages with concordant bioacoustic or morphological differentiation, thus almost certainly constituting distinct species that however have not yet been formally described[4]. We here include both described and confirmed candidate species and refer to all of them in the following as species.

Analysis of the mantellid radiation: adult morphological characters. Morphometric measurements were taken and morphological characters assessed in a total of 224 adult specimens preserved in the Zoological State Collection in Munich (ZSM), from specimens collected between the years 1998 and 2010. In most of the cases the specimens measured were males; females were measured for *Boophis arcanus, B. praedictus, Gephyromantis horridus, Guibemantis* sp. aff. *punctatus South, Mantidactylus charlotteae* Marojejy, *M. cowanii* and *Tsingymantis antitra* as no male specimens were available. For *Boophis lichenoides* several pictures from a specimen from the Museum of Comparative Zoology at Harvard University digital collection, were accessed online and used for character coding[5]. For six other species, *Blommersia angolafa, Boehmantis microtympanum, Gephyromantis plicifer, G. salegy, G. atsingy,* and *Spinomantis microtis* morphological information was gathered from the literature[6-10]. A total of 43 morphological characters for adult frogs were selected based on previous studies[11] and scored for all specimens . Data for four morphometric characters were based on original measurements by MV, completed with published information [6-9, 12-34] and transformed into categorical character states. Five characters included information about eye and head coloration patterns and were taken from the literature[35] as was maximum male snout-vent length (SVL)[36]. Those multistate characters that imply a continuum (i.e. the hand webbing which can have values form 0 to 4 according to the segment of the finger were it is originated), were considered as additive.

Analysis of the mantellid radiation: tadpole morphological characters. Several authors have studied and produced sets of morphological data for the larval phase in mantellids[37-47]. For the present study we built on these published data and prepared a novel dataset of more than 100 tadpole characters for 170 species of almost all the genera of the family, using previously defined characters (Supplementary Fig. 8). Continuous characters were transformed into categorical states as with adult characters.

Rates of phenotypic diversification: Effects of character coding. To identify rates of morphological evolution, we applied a Bayesian approach that requires a single test variable and therefore used variation in each first MDS variable as proxy for overall morphological

change. Since the number of morphological characters scored for tadpole life-history phase was higher than the number scored in adult frogs, we explored whether this number might influence the resulting posterior rates. For this purpose we randomly selected a number of 43 characters from the tadpole dataset and repeated *auteur* (Supplementary Table 9). We also explored whether the character coding scheme influenced resulting posterior rates, by removing all non-binary characters from the matrix and repeating auteur again with 44 binary coded characters (Supplementary Tables 8 and 9). The coding method or higher number of characters did not seem to influence estimated posterior rates, so that we proceeded with the original MDS based data sets for adult and tadpole morphology.

Detection of adaptive phenotypic optima: Additional methodological details. We estimated the numbers of different phenotypic optima (and selective regimes) occupied by adult and tadpole life-history phases by means of an Ornstein-Uhlenbeck process (OU). This model is an extension of the Brownian motion model in which a deterministic tendency toward adaptive optima is incorporated[48,49]. Four parameters regulate an OU model, namely: a Brownian rate parameter (σ^2), a rate of adaptation towards an optimum (α), and the optimum trait value (θ). We used the package *surface*[50] to explore how many different OU models (or selective regimes) could be fitted across the phylogeny using adult and tadpole traits independently (using the MDS variables described above). The fitting of the OU models by *surface* is conducted in a forward and a backward phase. This procedure allows the detection, not only of different selective regimes, but also of convergent regimes across the phylogeny[50]. For all analyses, in both forward and backward phases, we accepted all improvements of AICc values and allowed multiple compatible regimes to collapse during each step in the backward phase. Once the different selective regimes were defined for both adults and tadpole life history phases, these were visualized on the phylogeny, and by means of bivariate plots. For both adults and tadpoles, we compared the extent of convergence in phenotypic optima obtained to a null distribution using 100 replicates of nonconvergent OU models (Supplementary Fig. 7).

Correlation among single characters of adult and tadpole morphology. We searched for correlation between adult and tadpole morphology, accounting for correlations caused by the phylogenetic relationships among the different species of frogs. This was done using the option Multistate in BayesTraits[51] package. This program uses a continuous-time Markov model for the evolution of discrete multistate characters[52]. It allows to estimate rates of evolution of each character along the tree, and also to estimate the correlation between two characters comparing the likelihoods of a model that assumes dependent evolution and a model that assumes that the characters have evolved independently of each other along the given phylogeny.

Based on the guide tree[36], we compared each possible combination of an adult character and a tadpole character for two hypotheses, namely independent vs. correlated evolution of each two characters along the phylogeny. The logarithm of the likelihood ratio of the two models, L, was used as a test statistic[53]. To evaluate whether L gives significant evidence for correlation, we compared it to a distribution of L values simulated under the null hypothesis of independent evolution of the characters. For each pair of characters we generated 100 random matrices with the same dimensions and order of the characters as in our dataset. The character states were randomly generated based on the evolution rates estimated by BayesTraits for the original characters. We applied BayesTraits using the multistate option to each of the 100 simulated datasets in the same way as we did with the original matrix. Thus, we obtained a random distribution of L values that maintained the phylogenetic signal in each of the characters. The p-value in this test is then the relative rank of the original L among the simulated L values. As we performed this test not just once but for each character

pair, we had to correct for multiple testing. For this, we converted the *p*-values into False Discovery Rates (FDR) with the R package fdrtools[54]. All the simulations and comparisons were calculated using R.

92.5% of the pairwise comparisons between adult and tadpole morphological characters showed none or only a weak correlation (3937 out of 4255). Out of the 4255 possible comparisons between adult and tadpole characters, 424 obtained a *p*-value smaller than 0.05 (Supplementary Fig. 7). Its smallest *q*-value correspond to an FDR of 0.25, which means that we estimate that a quarter of these cases are type-I-errors and 318 character pairs evolved in a correlated way. This translates into 7.5% of all the performed comparisons co-evolving tightly enough to be detected by our methods. We therefore reject the null hypothesis of only random character correlations between phases, as more than 5% of the p-values were below 0.05. However, only a few of all possible characters co-evolve between adult and tadpole phases.

Supplementary Tables

Supplementary Table 1. List of genes associated with external morphology-associated Gene Ontologies that are expressed in different phases and combinations of these in *Xenopus laevis*. ALL – Genes expressed across all phases.

**Tadpole**	AMIGO1, AMPD2, APOB, COCH, COL14A1, IL10RA, PNPLA8, RGS18, SOBP, TMEM30C, VEPH1, AKT1, APOA4, ATP2B3, BTN2A2, CACNG2, CDHR1, COL13A1, CPNE7, CYP24A1, CYP46A1, CYTL1, EPYC, FAM126A, FOXN1, GNG2, HBD, IFITM5, KCTD7, LAMB3, MPZ, MYH3, NPHP4, PAPPA2, PRMT2, SCN8A, SERAC1, SLC4A8, SRPX2, SYT2, THIBZ, XAK-B
**Adult**	WDR35, RAB3GAP2, TRAPPC9, TGDS, SLC2A10, SCARF2, SLCO2A1, VPS33B, TTC8, RPGRIP1, TYR, TSC1, TNFRSF11B, VARS2, SYNGAP1, SH3TC2, RD3, PTK2B, SLC12A9, RGR, SLC7A14, SRD5A2, RMND1, TRY10, SYT14, TNF, SLC6A2, TMPRSS15, RDH5, SYCP3, RNF170, SLC24A1, SLC17A8, ZCCHC14, TPPP3, SYK, STAB2, TMEM19, ZWILCH, SP9, SEC22C, TMEM59L, SNX11, REM2, TNFRSF21, XKR4, YJEFN3, PTPRB, SUSD2, VCPIP1, RD3L, USP28, TACC1, SUSD4, PSMB9, SPA17, PSMG3, RGL2, TNFRSF9, ZNF536, CTSK, GLB1L, CYP19A1, LMNA, UROS, FAM20A, DDC, LRP2, LPL, UGT1A6, C4A, BLNK, TCAP, LIPC, PSTPIP1, CPS1, MTMR2, CAPN3, FHL1, CNTNAP1, RAB39B, MYBPC1, FCRL2, NFIL3, HYI, TNK2, IFT52, PLEKHA2, NTM, PLAT, FBXO8, STMN4, WWP2, CLYBL, DENND4B, GALNT13, IGLON5, BMP6, LIM2, CRYBB3, HOGA1, NR3C2, TMLHE, CTSF
**All**	ARSB, STAT1, TGM1, NFIX, SLC34A3, CYP4F22, EHHADH, HGD, KRT6, KRT79, KRT24, MLPH, PRPH2, ABCB1, ROM1, ABCA4, AGXT, EPHX1, AGT, HLA-DRB1, SAG, B2M, HSPD1, MYH13, EEF1A2, SGCE, SLC25A4, DMGDH, TNNT1, SERPINA3K, BGLAP, NTF3, FABP3, FKBP7, CADM4, DACH2, TINAGL1, SYBU, NLGN3, GC, ETNPPL, DCN, PTPRO, ZBTB1, PDGFRL, SERPINI1

Supplementary Table 2. Results from two-tailed Z-tests for differences in proportion of morphological feature formation-associated genes a) expressed across all phases (APG) and b) phase-specifically expressed genes (ASG adult-specific genes; TSG – tadpole-specific genes). Significance and z-levels are Bonferroni-adjusted for two tests per species ($\alpha = 0.017$; $z \geq 2.39$).

**Species, test**	**z-score**	**p**	**Lower 97% Conf. Interval**	**Upper 97% Conf. Interval**
Xenopus laevis				
APG / TSG	8.2487	≤0.0001	4.058	9.008
APG / ASG	11.6886	≤0.0001	6.400	10.190
Mantidactylus betsileanus				
APG / TSG	5.9551	≤0.0001	0.246	0.528
APG / ASG	2.6215	0.0088	0.015	0.159

Supplementary Table 3. Count of genes that are present in protein interaction subnetworks. Subnetworks denote phase-specific and morphology-associated genes that are functionally or phylogenetically connected between *Manidactylus betsileanus* and *Xenopus laevis*. Figures of these networks are depicted in Supplementary Figs 1 and 2. TAD – tadpole; ADU – adult.

**Phase**	**Subnetwork**	**Count of genes**
ADU	2	2
	8	3
	9	3
	12	3
	13	5
	14	32
TAD	15	2
	17	3
	20	5

Supplementary Table 4. Character states of all mantellid adult morphological characters used for analysis of phenotypic evolution.

**Character 1**. Femoral glands. State 0: Absent. State 1: Without central depression. State 2: With central depression.
**Character 2.** Snout shape in dorsal view. State 0: Semicircular. State 1: Nearly rounded. State 2: Rounded. State 3: Subovoid. State 4: subelliptical. State 5: Pointed. State 6: Truncate.
**Character 3.** Snout shape lateral. State 0: Rounded. State 1: Vertical. State 2: Obtuse. State 3: Acute. State 4: Acuminate. State 5: Protruding
**Character 4.** Canthus rostralis (CR) and loreal region (LR). State 0: CR very obtuse, LR concave. State 1: CR very obtuse, LR vertical or slightly concave. State 2: CR slightly marked; LR convex. State 3: CR rounded (indistinct), LR convex.
**Character 5.** Tympanum coloration. State 0: Indistinct. State 1: Same coloration as the rest of the head. State 2: Superior half of the tympanum coloured different from surrounding skin. State 3: Tympanum fully coloured differently from surrounding skin.
**Character 6.** Nuptial pads. State 0: Absent. State 1: Light-coloured on one finger. State 2: Light-coloured on two fingers. State 3: Dark-coloured on one finger. State 4: Dark coloured on two fingers.
**Character 7.** Relative length of fingers. State 0: Finger 1 < 2 < 4 < 3. State 1: Finger 2 < 4 < 1 < 3.
**Character 8.** Relative length of toes. State 0: Toe 1 < 2 < 3 < 5 < 4. State 1: Toe 1 < 2 < 5 < 3 < 4.
**Character 9.** Hand webbing. State 0: Absent. State 1: Present.
**Character 10.** Webbing formula on foot, forth toe (given as number of free phalanges not counting terminal phalanx). State 0: 4i(0). State 1: 4i(1). State 2: 4i(2). State 3: 4i(3). State 4: Webbing absent or rudimentary.
**Character 11.** Texture of dorsal skin. State 0: Areolate or smooth. State 1: Granular or tubercular. State 2: Dermal ridges (and/or reticulations).
**Character 12.** Texture of ventral skin. State 0: Coarsely aerolate. State 1: Areolate. State 2: Smooth.
**Character 13.** Relative hindlimb length (when adpressed along body, tibiotarsal articulation reaches.). State 0: To forelimb insertion, or tympanum, or eye center. State 1: To posterior eye corner, or nostril. State 2: To nostril, or snout tip, or beyound snout tip.
**Character 14.** Maximum male snout-vent length in mm. State 0: >13 and <26. State 1: >26 and <39. State 2: >39 and <52. State 3: >52 and <65. State 4: >65 and <78. State 5: >78 and <91. State 6: >91 and <104.
**Character 15.** Separation of lateral metatarsalia. State 0: Metatarsalia connected by muscular tissue. State 1: Metatarsalia separated (connected by webbing only).
**Character 16.** Terminal ending of fingers and toes. State 0: Not enlarged. State 1: Slightly enlarged. State 2: Enlarged semicircular or triangular discs.
**Character 17.** Frenal stripe. State 0: Absent. State 1: Present.
**Character 18.** Rostral stripe. State 0: Absent. State 1: Present.
**Character 19.** Interorbital markings. State 0: Absent. State 1: Transversal bar. State 2: One or several blotches. State 3: Longitudinal bar.
**Character 20.** Face mask. State 0: Absent. State 1: Present.
**Character 21.** Labial bars (alternating dark and light patches along lips). State 0: Absent. State 1: Present.
**Character 22.** Dorsal pattern. State 0: Light vertebral line, with otherwise uniform dorsal and lateral colour, or with a distinct colouration border between back and flanks, or with blotches. State 1: Light vertebral band, with otherwise uniform dorsal and lateral colour, or with dorsi-lateral bands, or with blotches. State 2: Sand-glass shaped marking, with otherwise uniform dorsal and lateral colour, or with blotches, or with dorsilateral bands, or with flecks. State 3: Chevron with otherwise uniform dorsal and lateral colour, or with transveral band, or with distinct coloration border between back and flanks. State 4: Dorsi-lateral bands with otherwise uniform dorsal and lateral colour, or with blotches or flecks. State 5: Uniform dorsal and lateral colour. State 6: Dorsally and/or laterally with flecks. State 7: Dorsally and/or laterally with blotches. State 8: Presence of a distinct colouration at the insertion of fore- and hindlimbs, with a V-shaped marking in the head, with or whitout blotches. State 9: Distinct colouration border between back and flanks, with otherwise uniform dorsal and lateral colour, or with blotches, or with flecks. State 10: One big transversal blotch, with otherwise uniform dorsal and lateral colour, or with distinct coloration border between back and flanks.
**Character 23.** Groin pattern of distinct light (often yellow) and black markings. State 0: Absent. State 1: Present.
**Character 24.** Axillary light-coloured marking. State 0: Absent. State 1: Present.
**Character 25.** Ventral pattern. State 0: Uniform. State 1: Throat dark grey or brown with pale flecks and pale medial streak. State 2: Narrow pale stripe from chin to thighs, transversally crossed by two pale lines. State 3: Chest and belly with black markings on pale grey venter, black spots on arms and legs, thighs dark gray, palmar and plantar surfaces dark grey with white tubercles. State 4: A few scattered spots. State 5: Presence of horse-shoe marking. State 6: Flecks evenly distributed on throat, chest, and belly. State 7: Dark markings around throat (collar). State 8: Dark markings on belly.
**Character 26.** Intercalary elements between ultimate and penultimate phalanges of fingers and toes. State 0: Absent. State 1: Present.
**Character 27.** Ventral skin pigmentation in life. State 0: Unpigmented transparent. State 1: Pigmented.
**Character 28.** Tibial glands. State 0: Absent. State 1: Present.
**Character 29.** Interocular tubercles. State 0: Absent. State 1: Present.
**Character 30.** Predominant dorsal color. State 0: Predominantly green. State 1: Predominantly brownish. State 2: Predominantly black-

yellow-red-orange (aposematic).
**Character 31.** Overall iris pattern. State 0: Horizontal. State 1: Uniform. State 2: Annular.
**Character 32.** Specific iris pattern. State 0: Horizontally contrasted. State 1: Horizontally divided. State 2: Densely reticulated. State 3: Horizontally striped. State 4: Uniform. State 5: Annular. State 6: Black eyes.
**Character 33.** Iris ornament. State 0: Absence. State 1: Presence.
**Character 34.** Iris periphery. State 0: Absence. State 1: Presence.
**Character 35.** Color of iris periphery. State 0: Indistinct. State 1: Blue. State 2: White. State 3: Yellow. State 4: Green. State 5: Bluish. State 6: Red.
**Character 36.** Adaptive functional coloration. State 0: Cryptic. State 1: Aposematic.
**Character 37.** Presence of teeth. State 0: Absence. State 1: Presence.
**Character 38.** Presence of rudimentary glands in females. State 0: Absence. State 1: Presence.
**Character 39.** Ratio head width / snout-vent length. State 0: > 0.325. State 1: > 0.362 and < 0.400. State 2: > 0.400 and < 0.437. State 3: > 0.437.
**Character 40.** Ratio head length / snout-vent length. State 0: > 0.364. State 1: > 0.403 and < 0.442. State 2: > 0.442 and < 0.481. State 3: > 0.481.
**Character 41.** Ratio eye diameter / snout-vent length. State 0: > 0.119. State 1: > 0.139 and < 0.159. State 2: > 0.159 and < 0.179. State 3: > 0.179.
**Character 42.** Ratio tympanum diameter / snout vent length (males). State 0: > 0.078. State 1: > 0.117 and < 0.155. State 2: > 0.155 and < 0.193. State 3: > 0.193.
**Character 43.** Heel tubercles. State 0: Absence. State 1: Presence.

Supplementary Table 5. Character states of all mantellid larval morphological characters used for analysis of phenotypic evolution. See Supplementary Fig. 8 for landmarks of measurements.

**Character 1.** Body shape: ratio body width (BW) vs. body height (BH). State 0: < 100% (compressed). State 1: > 100% (depressed).
**Character 2.** Body form in dorsal view. State 0: Ovoid. State 1: Elliptical. State 2: Rounded.
**Character 3.** Ratio body width (BW) vs. body length (BL). State 0: < 50%. State 1: 50-60%. State 2: 60-70%. State 3: >70%.
**Character 4.** Location of maximal body width (SBW), relative to body length (BL). State 0: In the proximal 1/5 of the body (<20%). State 1: In the proximal 1/5 to 2/5 of the body (20−40%). State 2: In the proximal 2/5 to 3/5 of the body (40−60%). State 3: In the proximal 3/5 to 4/5 of the body (60−80%). State 4: In the distal 1/5 of the body (>80%).
**Character 5.** Location of maximal body height (SBH), relative to body length (BL). State 0: In the proximal 1/5 of the body (<20%). State 1: In the proximal 1/5 to 2/5 of the body (20−40%). State 2: In the proximal 2/5 to 3/5 of the body (40−60%). State 3: In the proximal 3/5 to 4/5 of the body (60−80%). State 4: In the distal 1/5 of the body (>80%).
**Character 6.** Shape of snout in dorsal view. State 0: Pointed (sharp). State 1: Narrowly rounded. State 2: Broadly rounded. State 3: Flatly rounded. State 4: Rounded snout with protuberance.
**Character 7.** Shape of snout in lateral view. State 0: Pointed (sharp). State 1: Narrowly rounded. State 2: Broadly rounded. State 3: Flatly rounded.
**Character 8.** Ratio eye diameter (ED) vs. body length (BL). State 0: < 10% (small). State 1: 10−15% (moderately large). State 2: 15−20% (large). State 3: > 20% (very large).
**Character 9.** Position of eyes. State 0: Lateral. State 1: Dorsal.
**Character 10.** Location of eyes relative to body length (BL). State 0: In the 1/10 proximal of the body (< 10%). State 1: In the proximal 1/10 to 2/10 of the body (10−20%). State 2: In the proximal 2/10 to 3/10 of the body (20−30%). State 3: In the proximal 3/10 to 4/10 of the body (30−40%). State 4: In the proximal 4/10 to 5/10 of the body (40−46%). State 5: At mid-body (> 47%).
**Character 11.** Vertical position of eyes (EH), relative to body height (BH). State 0: < 20% (very low). State 1: 20−40% (low). State 2: 40−60% (moderately high). State 3: 60−80% (high). State 4: > 80% (very high).
**Character 12.** Orientation of eyes. State 0: Lateral. State 1: Dorsolateral. State 2: Dorsal.
**Character 13.** Ratio interocular diameter (IOD) vs. body width (BW). State 0: < 20% (very narrow). State 1: 20−40% (narrow). State 2: 40−60% (moderately wide). State 3: 60−80% (wide). State 4: > 80% (very wide).
**Character 14.** Eyes visibile from ventral view of tadpole. State 0: No. State 1: Yes.
**Character 15.** Ratio diameter of nares (ND) vs. body length (BL). State 0: < 1% (very small). State 1: 1−2% (small). State 2: 2−3% (moderately large). State 3: 3−4 % (large). State 4: > 4% (very large).
**Character 16.** Position of nares. State 0: Dorsal. State 1: Lateral. State 2: Ventral.
**Character 17.** Location of nares (RN relative to NP). State 0: Nearer to snout than to eyes (< 100%). State 1: Equidistant beteen snout and eyes (=100%). State 2: Nearer to eyes than to snout (> 100%).
**Character 18.** Vertical position of nares (NH), relative to body height (BH). State 0: < 20% (very low). State 1: 20−40% (low). State 2: 40−60% (moderately high). State 3: 60−80% (high). State 4: > 80% (very high).
**Character 19.** Vertical position of nares (NH) relative to position of eyes (NH relative to EH). State 0: below the level of the eye (< 96%). State 1: at the level of the eye (97−103%). State 2: above level of the the eye (> 104%).
**Character 20.** Orientation of narial opening in the horizontal plane. State 0: Anterior. State 1: Anterolateral. State 2: Lateral. State 3: Ventrolateral. State 4: Ventral.
**Character 21.** Orientation of narial opening in the vertical plane. State 0: Dorsal. State 1: Dorsolateral. State 2: Lateral. State 3: Ventrolateral. State 4: Ventral.
**Character 22.** Configuration of narial opening. State 0: At same level of surronding surface. State 1: Countersunk. State 2: Marked with a marginal rim.
**Character 23.** Form of narial opening. State 0: Rounded. State 1: Ovoid. State 2: Elliptical. State 3: Roughly triangular.
**Character 24.** Narial ornamentations. State 0: Nares not protruding. State 1: Nares protruding without ornamentation. State 2: Skin extension forming a tent-shaped flap over the nares. State 3: Small mediodorsal projection.
**Character 25.** Dark spot on the posterior side of nares. State 0: Absent. State 1: Present.
**Character 26.** Ratio nostril distance (IND) vs. interorbital distance (IOD). State 0: < 20% (very narrow). State 1: 20−40% (narrow). State 2: 40−60% (moderately wide). State 3: 60−80% (wide). State 4: > 80% (very wide).
**Character 27.** Spiracle type. State 0: Sinistral. State 1: Largely or fully ventral.
**Character 28.** Ratio spiracle length (SL) vs. body length (BL). State 0: < 10% (very short). State 1: 10−20% (short). State 2: 20−30% (moderately long). State 3: 30−40% (long). State 4: > 40% very long.
**Character 29.** Location of spiracle (SS) relative to body length (BL). State 0: In the proximal 1/5 of the body (<20%). State 1: In the proximal 1/5 to 2/5 of the body (20−40%). State 2: In the proximal 2/5 to 3/5 of the body (40−60%). State 3: In the proximal 3/5 to 4/5 of the body (60−80%). State 4: In the distal 1/5 of the body (>80%).
**Character 30.** Vertical location of spiracle (SH) relative to body height (BH). State 0: < 20% (very low). State 1: 20−40% (low). State 2: 40−60% (moderately high). State 3: 60−80% (high). State 4: > 80% (very high).

**Character 31.** Height of spiracle opening (SH), relative to the height of the point where the axis of the tail myotomes contacts the body (HAB). State 0: < 50% (at the height of the hindlimb insertion). State 1: 50−100% (below the height of the point where the axis of the tail myotomes contacts the body). State 2: 100% (at the height of the point where the axis of the tail myotomes contacts the body). State 3: > 100% (upper the height of the point where the axis of the tail myotomes contacts the body).
**Character 32.** Direction of spiracle opening. State 0: Dorsal. State 1: Posterodorsal. State 2: Posterior.
**Character 33.** Configuration of spiracle opening. State 0: Inner wall absent. State 1: Inner wall present as a slight ridge. State 2: Inner wall free from body, aperture opening posteriorly. State 3: Inner wall free from body and formed such that aperture opens laterally instead of posteriorly.
**Character 34.** Shape of spiracle opening. State 0: Rounded. State 1: Elliptical.
**Character 35.** Spiracle visibility from dorsal and ventral view. State 0: Visible only from dorsal view. State 1: Visible only from ventral view. State 2: Visible from dorsal and ventral views. State 3: Not visible from dorsal neither ventral view.
**Character 36.** Spiracle visibility from lateral view. State 0: Not recognizable. State 1: Weakly recognizable. State 2: Distinct.
**Character 37.** Ratio of vent tube length (VL) vs. body length (BL). State 0: < 5% (very short). State 1: 5−10% (short). State 2: 10−15% (moderately long). State 3: 15−20% (long). State 4: > 20% (very long).
**Character 38.** Vent tube in association with ventral fin. State 0: No. State 1: Yes.
**Character 39.** Configuration of vent tube opening if medial. State 0: Dextral. State 1: Medial, directly connected to body. State 2: Medial, connected to body by a a thin connecting skin. State 3: Medial, with lateral displacement. State 4: Medial, not associated with ventral fin. State 5: Medial, opening directly at the end of the body.
**Character 40.** Configuration of vent tube opening if dextral. State 0: Medial. State 1: Dextral with inner wall present. State 2: Dextral with inner wall absent.
**Character 41.** Visibility of intestine. State 0: Not visible due to pigmented ventral skin. State 1: Largely visible through transparent skin. State 2: Fully visible through translucent skin.
**Character 42.** Spiral-shape of intestine. State 0: Not visible. State 1: Regular. State 2: Irregular.
**Character 43.** Glands. State 0: Absent. State 1: Present.
**Character 44.** Ratio of tail length (TAL) vs. body length (BL). State 0: < 150% (tail very short). State 1: 150−200% (tail short). State 2: 200−250% (tail moderately long). State 3: 250−300% (tail long). State 4: > 300% (tail very long).
**Character 45.** Ratio of tail muscle width at the body-tail junction (TMW) vs. body width (BW). State 0: < 20% (very narrow). State 1: 20−40% (narrow). State 2: 40−60% (moderate). State 3: 60−80% (wide). State 4: > 80% (very wide).
**Character 46.** Ratio of tail muscle heigth at the body-tail junction (TMH) vs. body heigth (BH). State 0: < 20% (very low). State 1: 20−40% (low). State 2: 40−60% (moderate). State 3: 60−80% (high). State 4: > 80% (very high).
**Character 47.** Ratio of tail muscle height at mid-tail (TMHM) vs. tail heigth at mid tail (THM). State 0: < 20% (very low). State 1: 20−40% (low). State 2: 40−60% (moderate). State 3: 60−80% (high). State 4: 80−99% (very high). State 5: 100% (no fin at mid-tail).
**Character 48.** Ratio of tail heigth at midtail (TMHM) vs. maximal tail heigth (MTH). State 0: < 20% (very low). State 1: 20−40% (low). State 2: 40−60% (moderate). State 3: 60−80% (high). State 4: > 80% (very high).
**Character 49.** Ratio of maximal tail heigth (MTH) vs. body heigth (BH). State 0: < 96% (lower). State 1: 97-103% (same height). State 2: > 104% (higher).
**Character 50.** Ratio of tail muscle height (TMH) vs. maximal tail height (MTH). State 0: < 20% (very low). State 1: 20−40% (low). State 2: 40−60% (moderate). State 3: 60−80% (high). State 4: > 80% (very high).
**Character 51.** Ratio of tail height (TH) vs. body height (BH). State 0: < 96% (tail lower than body). State 1: 97-103% (tail and body of same height). State 2: > 104% (tail higher than body).
**Character 52.** Ratio of tail height at midtail (THM) vs. body height (BH). State 0: < 96% (tail lower than body). State 1: 97-103% (tail and body of same height). State 2: > 104% (tail higher than body).
**Character 53.** Ratio of tail height at midtail (THM) vs. maximal tail heigth (MTH). State 0: < 96% (THM lower than MTH). State 1: 97-100% (MTH=THM) height).
**Character 54.** Location of maximal tail heigth (DMTH) relative to TAL. State 0: In the proximal 1/5 of the tail (<20%). State 1: In the proximal 1/5 to 2/5 of the tail (20−40%). State 2: In the proximal 2/5 to 3/5 of the tail (40−60%). State 3: In the proximal 3/5 to 4/5 of the tail (60−80%). State 4: In the distal 1/5 of the tail (>80%).
**Character 55.** Tail muscle reaches end of tail. State 0: No. State 1: Yes.
**Character 56.** Visibility of lateral blood vessels in tail. State 0: Not visible. State 1: Visible in the proximal 1/4 of the tail. State 2: Visible in the first 1/2 of the tail. State 3: Visible in the first 3/4 of the tail. State 4: Visible in the whole tail.
**Character 57.** Height of the point where the axis of the tail myotomes contacts the body (HAB), relative to BH). State 0: < 20% (very low). State 1: 20−40% (low). State 2: 40−60% (moderately high). State 3: 60−80% (high). State 4: > 80% (very high).
**Character 58.** Axis of the body and axis of the tail myotomes. State 0: Parallel. State 1: Not parallel.
**Character 59.** Shape and vertical extension of dorsal fin (DF relative to TMHM). State 0: < 50% (very low). State 1: 50−100% (low). State 2: 100−150% (moderately high). State 3: 150−200% (high). State 4: > 200% (very high).
**Character 60.** Shape and vertical extension of ventral fin (VF relative to TMHM). State 0: < 50% (very low). State 1: 50−100% (low). State 2: 100−150% (moderately high). State 3: 150−200% (high). State 4: > 200% (very high).
**Character 61.** Ratio of vertical extension of dorsal fin (DF) vs. ventral fin (VF). State 0: < 96% (dorsal fin lower than ventral fin). State 1: 97-103% (vertical extension of dorsal fin similar to that of ventral fin). State 2: > 104% (dorsal fin higher than ventral fin).

**Character 62.** Location of the origin of dorsal fin. State 0: Anterior to dorsal body-tail junction. State 1: At dorsal body-tail junction. State 2: Between the dorsal body-tail and the proximal 1/4 of the tail. State 3: Between the proximal 1/4 and 1/2 of the tail. State 4: In the distal 1/2 of the tail.
**Character 63.** Location of the origin of ventral fin. State 0: Anterior to ventral body-tail junction. State 1: At ventral body-tail junction. State 2: Between the ventral body-tail junction and the proximal 1/4 of tail. State 3: Between the proximal 1/4 and 1/2 of the tail. State 4: In the distal 1/2 of the tail.
**Character 64.** Shape of tail tip. State 0: Pointed. State 1: Narrowly rounded. State 2: Broadly rounded.
**Character 65.** General kind of oral disc. State 0: Generalized. State 1: Enlarged. State 2: Reduced. State 3: Umbelliform. State 4: Highly modified. State 5: Reduced to a small opening.
**Character 66.** Emargination of oral disc. State 0: Emarginated. State 1: Not emarginated. State 2: Not applicable.
**Character 67.** Width of labia. State 0: Not applicable. State 1: Upper labium is wider than lower labium. State 2: Lower labium is wider than upper labium. State 3: The two labia have the same width.
**Character 68.** Ratio of oral disc width (ODW) vs. body width (BW). State 0: < 20% (oral disc very small). State 1: 20−40% (small). State 2: 40−60% (moderately large). State 3: 60−80% (large). State 4: 80−100% (very large). State 5: > 100% (extremely large).
**Character 69.** Shape of upper labium and adjacent part of body wall. State 0: Upper labium is a continuation of snout. State 1: Anterior margin of upper labium is separated by a shallow groove. State 2: Anterior margin of upper labium is separated by a deep groove (the entire margin is free from the snout). State 3: Umbrella-shaped oral disc. State 4: Oral disc has no connection with snout.
**Character 70.** Position of oral disc. State 0: Ventral. State 1: Anteroventral. State 2: Subterminal. State 3: Dorsal.
**Character 71.** Orientation of oral disc. State 0: Ventral. State 1: Anteroventral. State 2: Anterior. State 3: Anterodorsal. State 4: Dorsal.
**Character 72.** Visibility of oral disc in dorsal view. State 0: Not visible. State 1: Visible.
**Character 73.** Oral disc papillae. State 0: Absent. State 1: Present.
**Character 74.** Size of oral disc papillae. State 0: No papillae. State 1: Small-sized papillae. State 2: Moderately-sized papillae. State 3: Large papillae.
**Character 75.** Length of marginal papillae on oral disc. State 0: Absent. State 1: < 0.1 mm (very short). State 2: 0.1−0.2 mm (short). State 3: 0.2−0.3 mm (moderately long). State 4: > 0.3−0.4 mm (long). State 5: > 0.4 mm (very long).
**Character 76.** Length of submarginal papillae on oral disc. State 0: Absent. State 1: < 0.1 mm (very short). State 2: 0.1−0.2 mm (short). State 3: 0.2−0.3 mm (moderately long). State 4: > 0.3−0.4 mm (long). State 5: > 0.4 mm (very long).
**Character 77.** Form of oral disc papillae. State 0: No papillae. State 1: Suctorial type. State 2: Conical. State 3: Rounded. State 4: Elongated.
**Character 78.** Form of terminal tip of oral disc papillae. State 0: No papillae. State 1: Normal (rounded). State 2: Rounded with protuberance. State 3: Pointed. State 4: Marginal papillae rounded and submarginal papillae pointed.
**Character 79.** Number of rows of marginal papillae on oral disc. State 0: No papillae. State 1: Single. State 2: Double. State 3: Triple.
**Character 80.** Gap(s) in rows of marginal papillae on oral disc. State 0: No papillae. State 1: Dorsal and ventral gap. State 2: Dorsal gap only. State 3: Ventral gap only. State 4: Complete (no gap).
**Character 81.** Number of marginal papillae on oral disc. State 0: No papillae. State 1: < 50 (very few papillae). State 2: 50−100 (few). State 3: 100−200 (moderate number). State 4: 200−300 (many). State 5: > 300 (very many).
**Character 82.** Number of submarginal papillae on oral disc. State 0: no papillae. State 1: < 50 (very few). State 2: 50−100 (few). State 3: 100−200 (moderatelly many). State 4: 200−300 (many). State 5: > 300 (very many).
**Character 83.** Position of submarginal papillae on lower labium of oral disc. State 0: No papillae. State 1: Papillae positioned only ventrally. State 2: Papillae positioned only laterally. State 3: Papillae positioned ventrolaterally. State 4: Papillae positioned ventrolaterally and laterally. State 5: Papillae positioned ventrally, ventrolaterally, and laterally.
**Character 84.** Position of submarginal papillae on upper labium of oral disc. State 0: No papillae. State 1: Papillae positioned only dorsally. State 2: Papillae positioned only laterally. State 3: Papillae positioned dorsolaterally. State 4: Papillae positioned dorsolaterally and laterally. State 5: Papillae positioned dorsally, dorsolaterally, and laterally.
**Character 85.** Ratio of width of dorsal gap in rows of papillae (DG) vs. oral disc width (ODW). State 0: Gap absent. State 1: < 20% (gap very narrow). State 2: 20−40% (gap narrow). State 3: 40−60% (gap moderately wide). State 4: 60−80% (gap wide). State 5: > 80% (gap very wide).
**Character 86.** Ratio of width of dorsal gap in rows of papillae (VG) vs. oral disc width (ODW). State 0: Gap absent. State 1: < 20% (gap very narrow). State 2: 20−40% (gap narrow). State 3: 40−60% (gap moderately wide). State 4: 60−80% (gap wide). State 5: > 80% (gap very wide).
**Character 87.** Visibility of oral disc papillae in dorsal view. State 0: No papillae. State 1: Not visible. State 2: Visible.
**Character 88.** Keratodonts (labial teeth). State 0: Absent. State 1: Present.
**Character 89.** Number of ranges of keratodonts per labium . State 0: No keratodonts. State 1: 1 range of keratodonts. State 2: 2 ranges of keratodonts.
**Character 90.** Number of divided upper keratodont rows. State 0: No keratodonts or no divided upper row. State 1: 1 divided upper row. State 2: 2 divided upper rows. State 3: 3 divided upper rows. State 4: 4 divided upper rows. State 5: > 5 divided upper rows.
**Character 91.** First divided upper row. State 0: no keratodont or no divided upper row. State 1: Row A1. State 2: Row A2. State 3: Row A3. State 4: Row A4. State 5: Row A5 or higher number.
**Character 92.** Number of undivided upper keratodont rows. State 0: No keratodonts or no undivided upper row. State 1: 1 undivided upper row. State 2: 2 undivided upper rows. State 3: 3 undivided upper rows. State 4: 4 undivided upper rows. State 5: 5 or more undivided upper

rows.
**Character 93.** Number of divided lower keratodont row. State 0: No keratodonts. State 1: No divided lower keratodont rows. State 2: 1 divided lower keratodont row. State 3: 2 divided lower keratodont rows. State 4: 3 divided lower keratodont rows.
**Character 94.** First undivided lower row. State 0: No keratodonts. State 1: No undivided lower row. State 2: P1 is first divided lower keratodont row. State 3: P2 is first divided lower keratodont row. State 4: P3 is first divided lower keratodont row.
**Character 95.** Number of undivided lower keratodont rows. State 0: No keratodont. State 1: No undivided lower keratodont rows. State 2: 1 undivided lower keratodont rows. State 3: 2 undivided lower keratodont rows. State 4: 3 undivided lower keratodont rows.
**Character 96.** Keratodont length. State 0: No keratodonts. State 1: < 0.05 mm (very short). State 2: 0.05−0.10 mm (short). State 3: 0.10−0.20 mm (moderately long). State 4: 0.20−0.30 mm (long). State 5: > 0.30 mm (very long).
**Character 97.** Ratio of the length of keratodont row A1 vs. ODW. State 0: No keratodonts. State 1: < 20% (row A1 very short). State 2: 20−40% (row A1 short). State 3: 40−60% (row A1 moderately long). State 4: 60−80% (row A1 long). State 5: > 80% (row A1 very long).
**Character 98.** Distal keratodont size. State 0: No keratodonts. State 1: Same length as those in the middle of the row. State 2: Slightly shorter. State 3: Shorter.
**Character 99.** General arrangement of keratodont rows. State. State 0: No keratodonts. State 1: Intact. State 2: Scattered.
**Character 100.** Alignment of keratodont rows. State 0: No keratodonts. State 1: Regular. State 2: Irregular.
**Character 101.** Lower keratodont rows forming a chevron. State 0: No keratodonts. State 1: No. State 2: Yes.
**Character 102.** Ratio of the length of the gap at the first upper interrupted row (A2 gap) vs. row length (A2). State 0: No gap. State 1: < 20% (very narrow). State 2: 20−40% (narrow). State 3: 40−60% (moderately wide). State 4: 60−80% (wide). State 5: > 80% (very wide).
**Character 103.** Arrangement of keratodont row P1. State 0: No keratodonts. State 1: Not interrupted. State 2: Interrupted.
**Character 104.** Space between marginal papillae and keratodont rows. State 0: No keratodonts or no papillae. State 1: Reduced space (papillae and keratodonts almost in contact). State 2: Wide space (distinct separation).
**Character 105.** Type of jaw sheath. State 0: Jaw sheath absent. State 1: Jaw sheath reduced. State 2: Jaw sheath generalized (typical, with smooth surface). State 3: Jaw sheath ribbed (giving the appearance of vertical bars). State 4: Jaw sheath transformed into long spikelike derivatives. State 5: Jaw sheath enlarged, *Mantella cowani*-like.
**Character 106.** Strength of jaw sheaths. State. State 0: Absent or not applicable. State 1: Jaw sheaths weakly expressed, soft. State 2: Jaw sheaths strong. State 3: Jaw sheaths very strong.
**Character 107.** Serration of jaw sheaths. State 0: No jaw sheaths or not applicable. State 1: Jaw sheaths with smooth edge. State 2: Jaw sheaths with rounded serrations. State 3: Jaw sheaths with pointed serrations. State 4: Jaw sheaths with finely pointed serrations. State 5: Jaw sheaths with hypertrophied serrations.
**Character 108.** Ratio of jaw sheath width (JW) vs. oral disc width (ODW). State 0: No jaw sheaths. State 1: < 20% (jaw sheaths very narrow). State 2: 20−40% (jaw sheaths narrow). State 3: 40−60% (jaw sheaths moderately wide). State 4: 60−80% (jaw sheaths wide). State 5: > 80% (jaw sheaths very wide).
**Character 109.** Presence of upper jaw sheath. State 0: Always present. State 1: Vestigial, sometimes absent. State 2: Always absent or not applicable. State 3: Highly modified.
**Character 110.** Width of upper jaw sheath medial convexity (MCL) relative to jaw sheath width (JW). State 0: No upper jaw sheath or not applicable. State 1: < 10 % (very narrow). State 2: 10−20 % (narrow). State 3: 20−30 % (moderately wide). State 4: 30- 40 % (wide). State 5: > 40 % (very wide).
**Character 111.** Shape of upper jaw sheath medial convexity. State 0: Upper jaw sheath absent. State 1: Medial convexity wide and rounded. State 2: Medial convexity narrow and rounded. State 3: Medial convexity wide and pointed. State 4: Medial convexity narrow and pointed. State 5: Medial convexity elongated.
**Character 112.** Keratinization of upper jaw sheath. State 0: No upper jaw sheaths. State 1: Not keratinized. State 2: Partially keratinized. State 3: Fully keratinized.
**Character 113.** Keratinization of lower jaw sheath. State 0: No lower jaw sheath. State 1: Not keratinized. State 2: Partially keratinized. State 3: Fully keratinized.
**Character 114.** Shape of lower jaw sheath. State 0: No lower jaw sheath. State 1: Not applicable. State 2: U-shaped. State 3: V-shaped.
**Character 115.** Visibility of lower jaw sheath visibility. State 0: No lower jaw sheath. State 1: Lower jaw sheath visible, not hidden by upper jaw sheath. State 2: Lower jaw sheath partially hidden by upper jaw sheath. State 3: Lower jaw sheath totally hidden by upper jaw sheath.
**Character 116.** Pigmentation pattern on body. State 0: Uniform. State 1: Spotted to blotched. State 2: Reticulated.
**Character 117.** Lateral space (probable lymphatic interstitial lacunes) under the skin. State 0: Absent. State 1: Weakly recognizable. State 2: Distinct.

Supplementary Table 6. Summary statistics for selective regime shifts inferred with SURFACE for mantellid morphological evolution. k–number of regime shifts, counting the basal regime as 1; kprime–number of regimes, some of which may be reached by multiple shifts; deltak–a measure of convergence; c–the number of shifts to convergent regimes (another measure of convergence); kprime_conv = the number of convergent regimes shifted to multiple times; kprime_nonconv = the number of nonconvergent regimes only shifted to once.

**Data set**	**k**	**kprime**	**deltak**	**c**	**kprime_conv**	**kprime_nonconv**	**p-value**
Adult phase	18	14	4	8	4	10	0.54
Tadpole phase	19	13	6	11	5	8	0.15

Supplementary Table 7. Results from selective optima analysis for the first two multidimensional scaling variables of mantellid adult and tadpole phenotype.

**I Adult phase**	**MDS Var1**	**MDS Var2**
alpha	0.1013475	0.2237273
phylogenetic_half_life	6.83931	3.098179
sigma_squared	0.001771307	0.016639991
optimum 1	0.54912534	-0.20591444
optimum 2	-0.26745187	0.078756978
optimum 3	0.21406944	-0.158158409
optimum 4	-0.05080339	0.342962803
optimum 5	-0.59144304	-0.213658551
optimum 6	-0.78322792	0.206943778
optimum 7	0.18749578	-0.008451292
optimum 8	0.6054657	0.306652101
optimum 9	-0.50364898	0.177636012
optimum 10	0.65790743	-0.156100889
optimum 11	-0.67677628	-0.597791399
optimum 12	-0.27917149	-0.46671403
optimum 13	0.46572214	0.182164685
optimum 14	-0.43340705	0.403314117
**II Tadpole phase**	**MDS Var1**	**MDS Var2**
alpha	12.04717394	3.86608626
phylogenetic_half_life	0.05753608	0.17928911
sigma_squared	0.283764407	0.153525932
optimum 1	0.39592	0.01564
optimum 2	-1.497	-0.281
optimum 3	-1.449	-0.205
optimum 4	-1.127625	0.296875
optimum 5	-0.8445	-0.286
optimum 6	-0.105875	-0.85625
optimum 7	0.03166667	0.2077037
optimum 8	0.10071429	-0.3778571
optimum 9	-0.074	0.1725
optimum 10	0.328	-0.574
optimum 11	0.22704	0.049
optimum 12	-1.508	0.099
optimum 13	0.044	0.546

Supplementary Table 8. Character states of 45 selected larval morphological characters used for analysis of phenotypic evolution under an alternative (binary) coding scheme, used to explore possible effects of coding scheme and number of characters on differences in rates of morphological evolution between adult and tadpole mantellid frogs (see Supplementary Tables 9 and 10). Character numbers and partly numbering of characters states correspond to those in Supplementary Table 5 for better comparability.

**Character 1.** Body shape: ratio body width (BW) vs. body height (BH). State 0: < 100% (compressed). State 1: > 100% (depressed).
**Character 3.** Ratio body width (BW) vs. body length (BL). State 0: < 60%. State 1: >60%
**Character 6.** Shape of snout in dorsal view. State 0: Pointed or narrowly rounded. State 2: Broadly or flatly rounded.
Character 6a: Snout protuberance 0 absent, 1 present
**Character 8.** Ratio eye diameter (ED) vs. body length (BL). State 0: < 15% (small). State 2: >15−20% (large or very large).
**Character 12.** Orientation of eyes. State 0: Lateral. State 1: Dorsolateral. State 2: Dorsal.
**Character 16.** Position of nares. State 0: Dorsal. State 1: Lateral.
**Character 27.** Spiracle type. State 0: Sinistral. State 1: Largely or fully ventral.
**Character 38.** Vent tube in association with ventral fin. State 0: No. State 1: Yes.
**Character 41.** Visibility of intestine. State 0: Not visible due to pigmented ventral skin. State 1: Largely or fully visible through transparent or translucent skin.
**Character 42.** Spiral-shape of intestine. State 1: Regular. State 2: Irregular.
**Character 43.** Glands. State 0: Absent. State 1: Present.
**Character 44.** Ratio of tail length (TAL) vs. body length (BL). State 0: < 200% (tail short or very short). State 2: >200−250% (tail long or very long).
**Character 47.** Ratio of tail muscle height at mid-tail (TMHM) vs. tail heigth at mid tail (THM). State 0: < 40% (low or very low). State 2: >40% (moderate height to absent fin).
**Character 49.** Ratio of maximal tail heigth (MTH) vs. body heigth (BH). State 0: < 96% - 103% (lower or same height). State 2: > 104% (higher).
**Character 55.** Tail muscle reaches end of tail. State 0: No. State 1: Yes.
**Character 56.** Visibility of lateral blood vessels in tail. State 0: Not visible or only in the proximal 1/4 of the tail. State 2: Visible in the first 1/2 of the tail or more
**Character 58.** Axis of the body and axis of the tail myotomes. State 0: Parallel. State 1: Not parallel.
**Character 59.** Shape and vertical extension of dorsal fin (DF relative to TMHM). State 0: < 50% to 100% (low to very low). State 2: >100% (moderately to very high).
**Character 60.** Shape and vertical extension of ventral fin (VF relative to TMHM). State 0: < 50% to 100% (very low to low). State 2: >100% (moderately to very high).
**Character 64.** Shape of tail tip. State 0: Pointed. State 1: Narrowly rounded to broadly rounded.
**Character 65.** General kind of oral disc. State 0: Generalized. State 1: Derived
**Character 65a.** Oral disc distinctly enlarged. State 0: No. State 1: Yes.
**Character 65b.** Oral disc visible, not reduced. State 2: Reduced or reduced to small opening.
**Character 65c.** Oral disc umbelliform. State 0: No. State 3: Yes
**Character 66.** Emargination of oral disc. State 0: Emarginated. State 1: Not emarginated.
**Character 68.** Ratio of oral disc width (ODW) vs. body width (BW). State 0: < 40% (oral disc small to very small). State 2: >40% (moderately to very large).
**Character 70.** Position of oral disc. State 0: Ventral or anteroventral. State 2: Subterminal or dorsal
**Character 73.** Oral disc papillae. State 0: Absent. State 1: Present.
**Character 74.** Size of oral disc papillae. State 1: Small-sized papillae. State 2: Moderately-sized or large papillae.
**Character 80.** Gap(s) in rows of marginal papillae on oral disc. State 1: With dorsal gap. State 3: With ventral gap only, or without gap
**Character 81.** Number of marginal papillae on oral disc. State 1: < 50 to 100 (very few or few papillae). State 3: >100 (moderate to many)
**Character 82.** Number of submarginal papillae on oral disc. State 1: < 50 to 100 (few or very few). State 3: >100−200 (moderate to many)
**Character 88.** Keratodonts (labial teeth). State 0: Absent. State 1: Present.

**Character 89.** Number of ranges of keratodonts per labium State 1: 1 range of keratodonts. State 2: 2 ranges of keratodonts.
**Character 91.** First divided upper row. State 1: Row A1 or A2. State 3: Row A3 or higher number.
**Character 93.** Number of divided lower keratodont row. State 2: 1 divided lower keratodont row. State 3: 2 or 3 divided lower keratodont rows.
**Character 96.** Keratodont length. State 1: < 0.05 to 0.10 mm (short or very short). State 3: >0.10−0.20 mm (moderately to very long).
**Character 101.** Lower keratodont rows forming a chevron. State 1: No. State 2: Yes.
**Character 112.** Keratinization of upper jaw sheath. State 1: Not or partially keratinized. State 3: Fully keratinized.
**Character 113.** Keratinization of lower jaw sheath.State 1: Not or partially keratinized. State 3: Fully keratinized.
**Character 116.** Pigmentation pattern on body. State 0: Uniform. State 1: Spotted to blotched or reticulated
**Character 116a.** Presence of reticulated pattern. State 0: Absent. State 2: Present.
**Character 117.** Lateral space (probable lymphatic interstitial lacunes) under the skin. State 0: Absent. State 1: Weakly recognizable to distinct.

Supplementary Table 9. Mean rates of morphological evolution in adult and tadpole life-history phases of the Mantellidae, obtained from different analyses with *auteur* package. Main analyses as used in Fig. 1 are those with N=207 tips, whereas for comparisons, two tips were excluded. The jackknifed data set of tadpoles was obtained by randomly excluding characters from the original 117 characters to obtain a matrix of 43 characters (as in the adult data set). The tadpole recoded data were obtained from the 44 binary-coded characters in Supplementary Table 8. Independent from coding scheme and number of characters in the underlying matrix, average rates of tadpoles are much lower than those of adults, suggesting that larval morphological evolution proceeds at a faster rate in these anurans instead of being an artifact of higher number of characters or character coding method.

	**N characters**	**N branches**	**Valid N**	**Mean**	**Minimum**	**Maximum**	**Std.Dev.**
Adults	43	221	205	0.213095	0.043173	0.339238	0.067024
Adults	43	223	207	0.212269	0.047005	0.324864	0.06597
Tadpoles	117	221	205	0.787647	0.075802	2.171042	0.277698
Tadpoles	117	223	207	0.765757	0.105502	2.576927	0.282486
Tadpoles (characters jackknifed)	43	221	205	0.873654	0.846038	1.087608	0.030246
Tadpoles (characters recoded)	44	221	205	0.701315	0.688001	1.301289	0.054259

Supplementary Table 10. Correlation analysis of rates per branch in four data sets. The rates obtained from the resampled and the recoded tadpole data sets are both correlated with those from the original tadpole data sets based on 117 characters, whereas none of the tadpole data sets shows a correlation with the adult morphological rates, as in the original data sets.

	**Tadpoles**	**Tadpoles recoded**	**Tadpoles resampled**
Adults	0.0731 (P=0.281)	0.1167 (P=0.084)	0.0600 (P=0.376)
Tadpoles	NA	***-0.3090 (P=0.000) ****	***0.2072 (P=0.002)****

Supplementary Figures

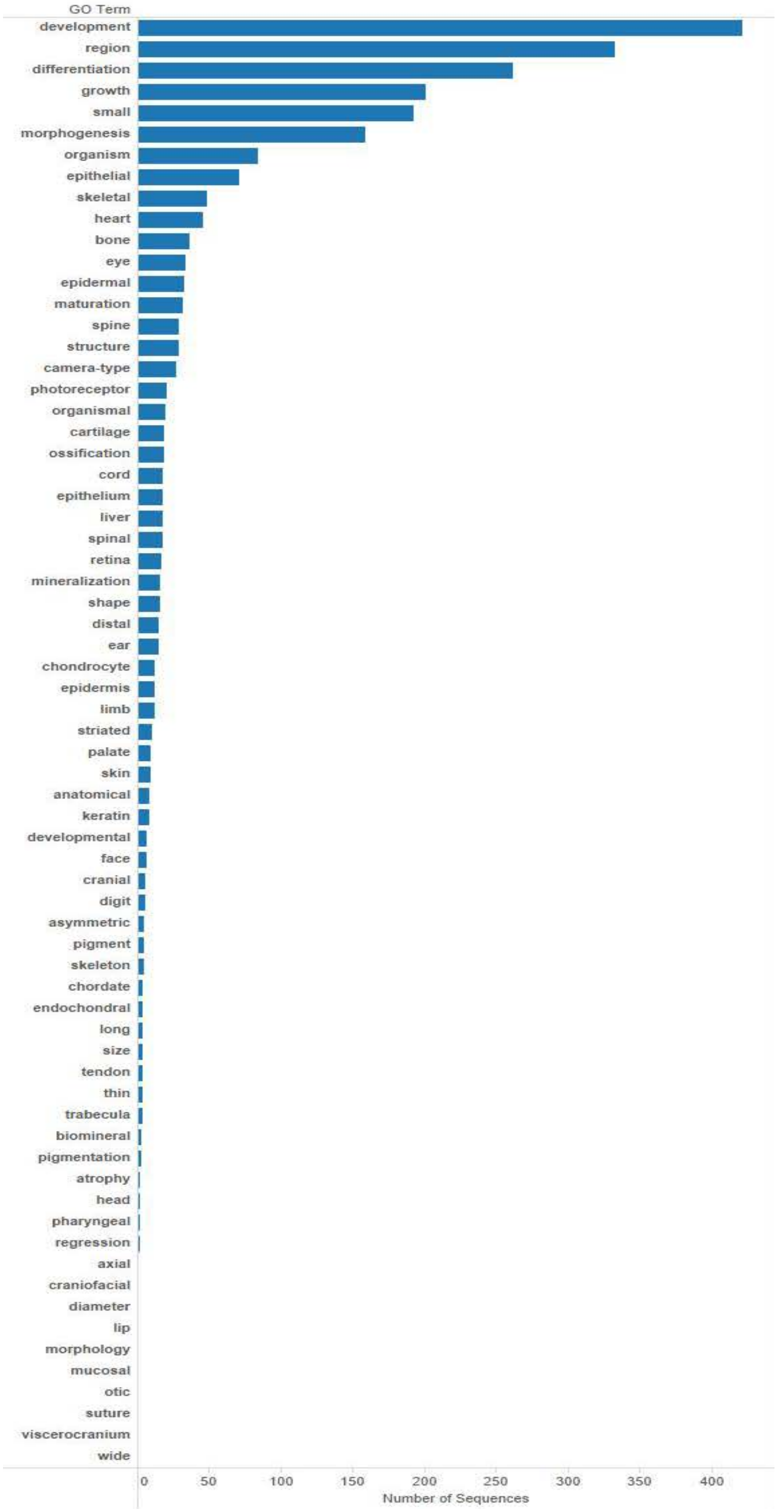

Supplementary Figure 1. Morphology-associated GO terms and number of associated transcripts in *Mantidactylus betsileanus* transcriptomes.

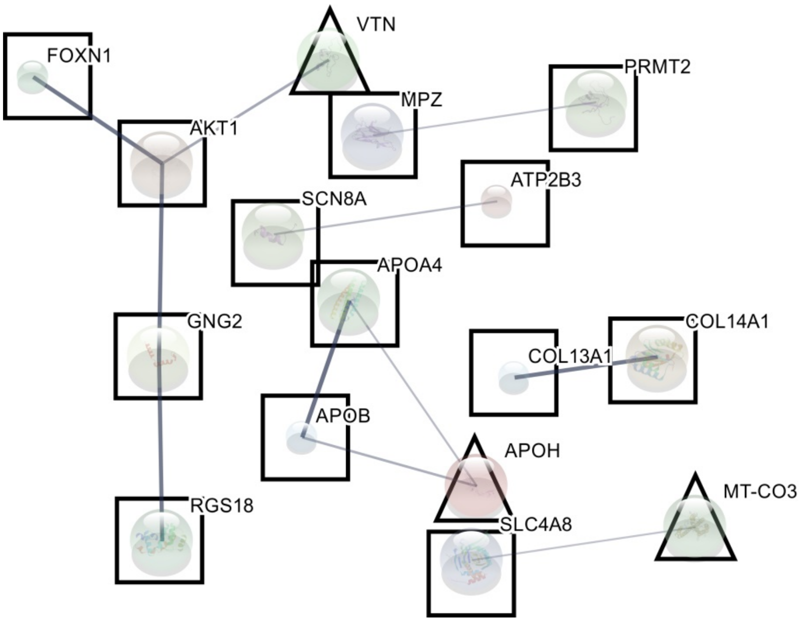

Supplementary Figure 2. Subnetworks representing protein-protein interactions among phenotype-associated genes of *Mantidactylus betsileanus* overexpressed in the tadpole phase, and of phase-specific genes associated with morphology in *Xenopus laevis*. Networks generated with STRING database (confidence view, human-based information on gene interactions). Boxes denote genes obtained from *Xenopus laevis* dataset; triangles denote genes obtained from *Mantidactylus betsileanus* dataset. Functional interactions were not significantly higher than expected, or significantly enriched with specific GO terms.

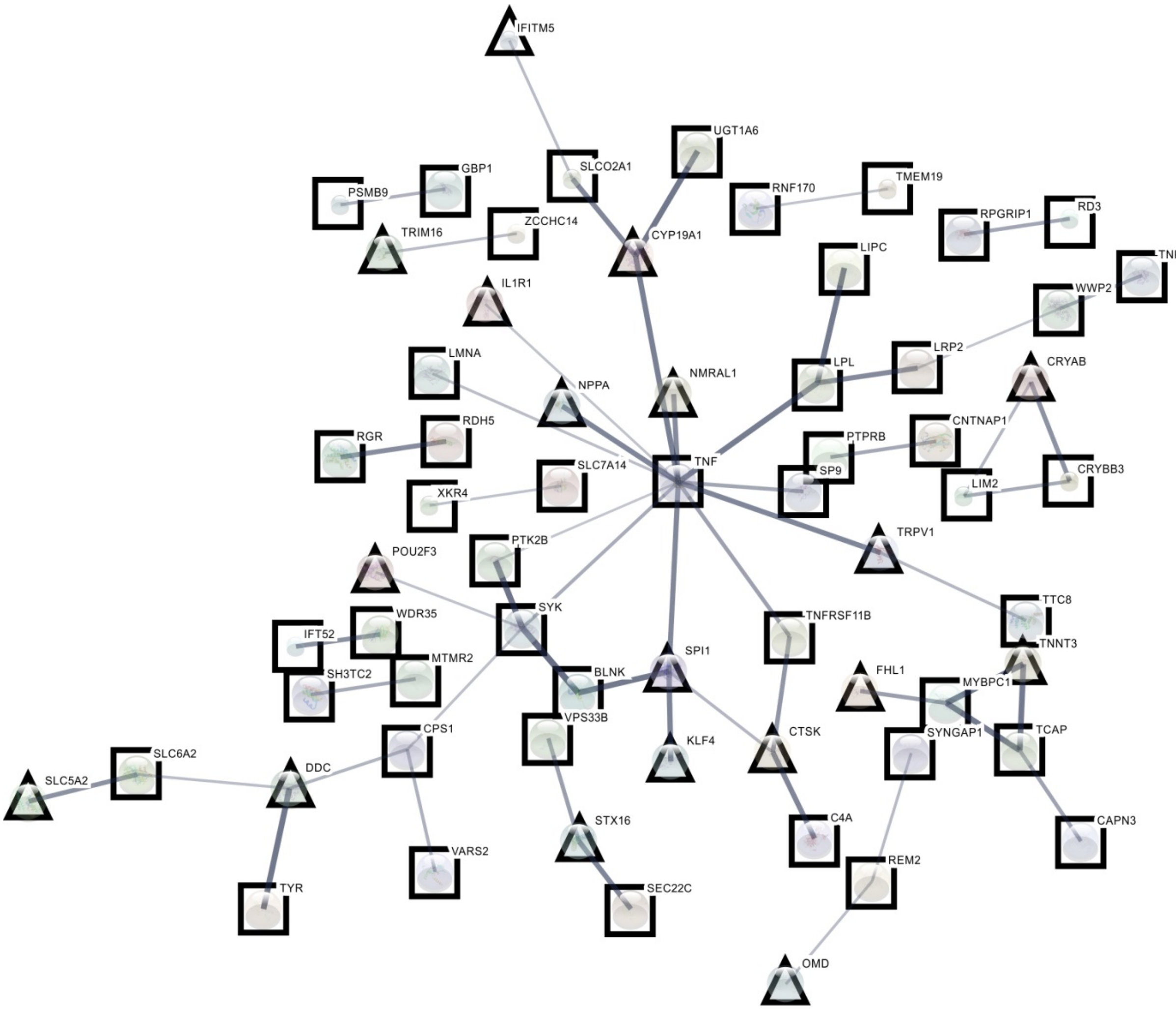

Supplementary Figure 3. Subnetworks representing protein-protein interactions among phenotype-associated genes of *Mantidactylus betsileanus* overexpressed in the adult phase (post-metamorphosis), and of phase-specific genes associated with morphology in *Xenopus laevis*. Networks generated with STRING database (confidence view, human-based interactions), boxes denote gene obtained from *Xenopus laevis* dataset; triangles denote genes obtained from *Mantidactylus betsileanus* dataset. Functional interactions were significantly higher than expected, and significanrtly enriched with KEGG pathway “osteoclast differentiation.

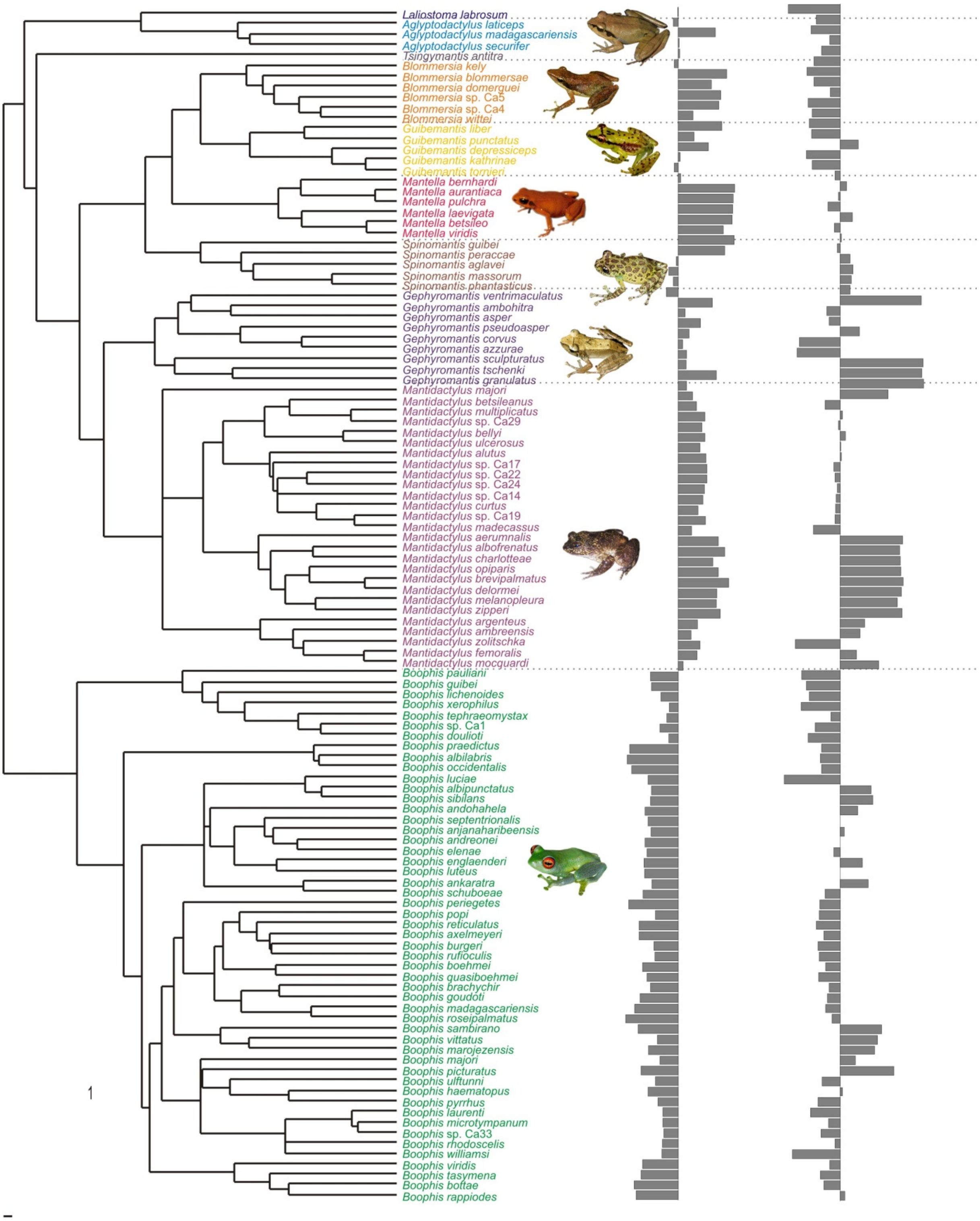

Supplementary Figure 4. Molecular timetree of mantellid species for which both tadpole and adult morphological data are available[36]. Species are colored according to their genus-level classification. Adult and tadpole morphology summary variables show scores for each species derived from a multidimensional scaling analysis (not phylogenetically corrected) of the respective data set. Inset photos show representative species of some genera.

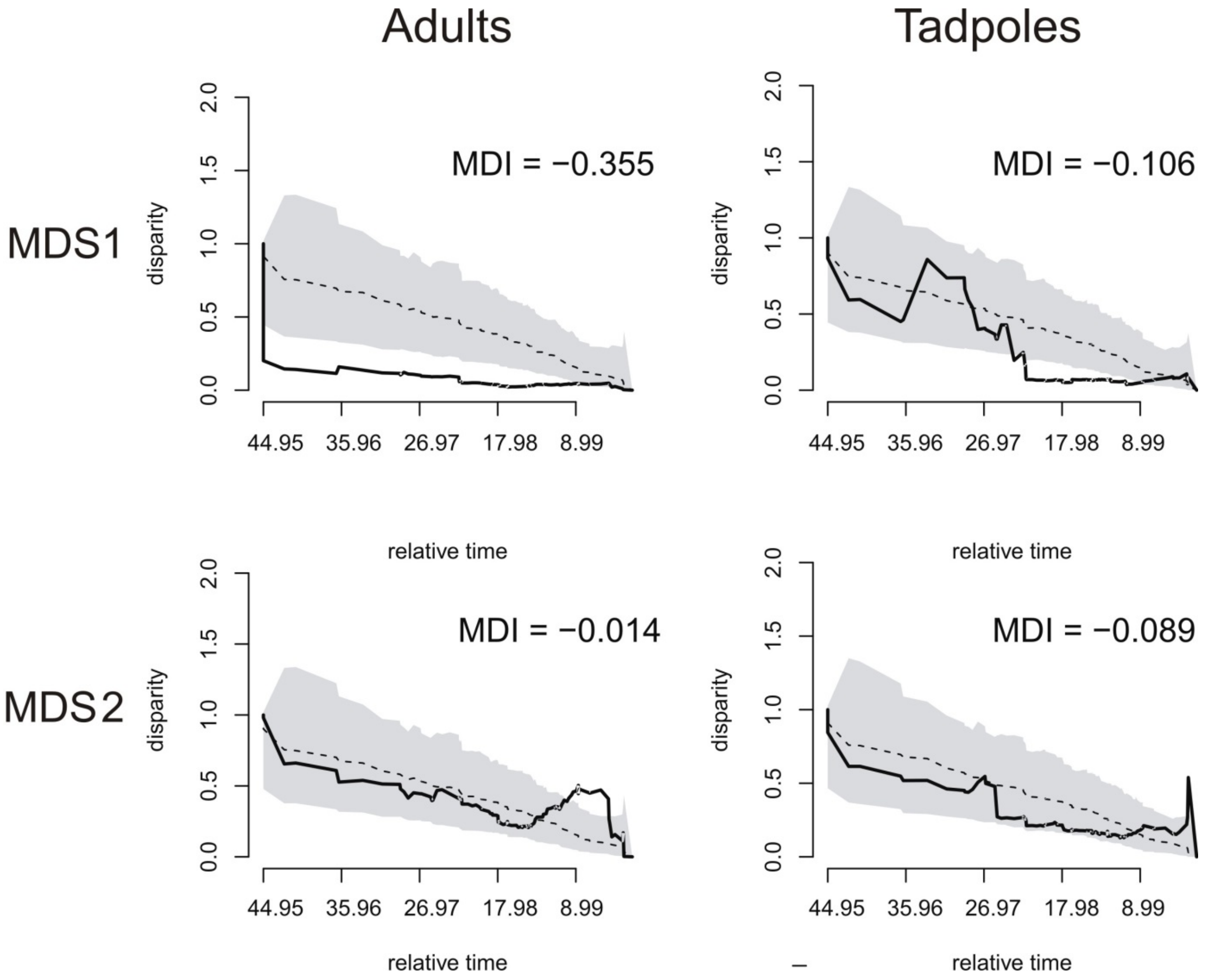

Supplementary Figure 5. Disparity through time (DTT) plots visualizing the dynamics of phenotypic diversification in adults and tadpoles of Mantellidae, for the two first MDS variables derived from the morphological data. The dashed line indicates the median subclade DTT based on 10,000 simulations of character evolution under Brownian motion, on the mantellid phylogeny. The grey shaded area represents the 95% DTT interval for the simulated data.

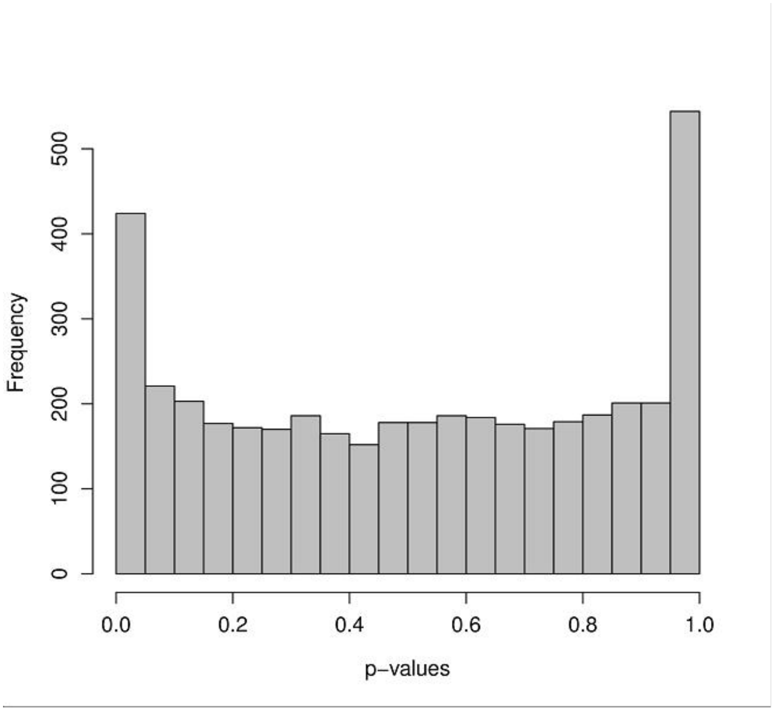

Supplementary Figure 6. Histogram of p-values obtained for the 4255 pairwise comparisons between evolutionary rates of all adult and tadpole characters. 7.5% of pairwise comparisons between tadpole and adult characters were significantly correlated (based on likelihood ratio test statistic, and after correction for false discovery rates; see Supplementary Methods). As the value is above 5%, this suggests that the rates of change in a few tadpole and adult characters are correlated.

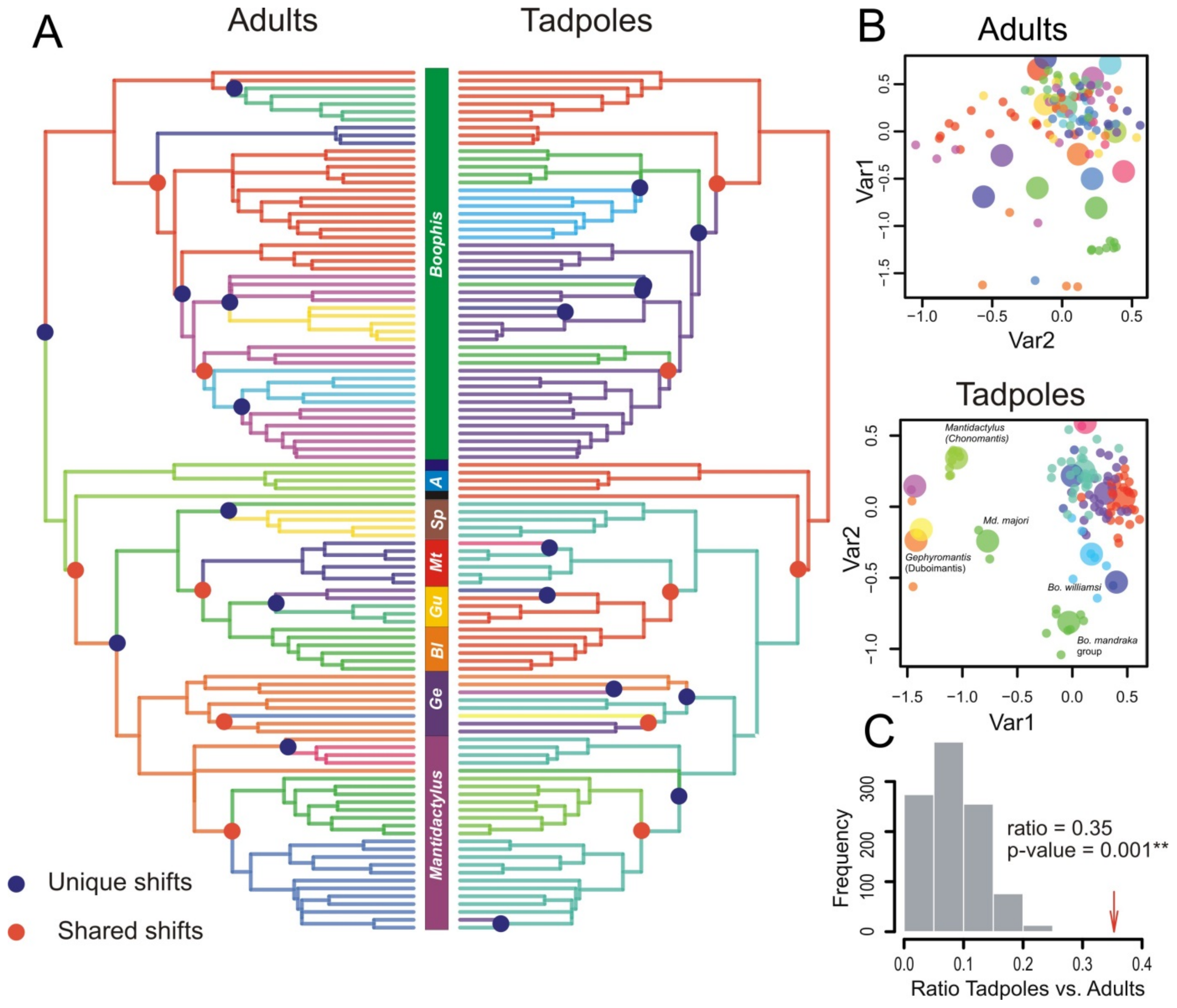

Supplementary Figure 7. Selective optima for mantellid tadpole and adult phenotypes mapped on molecular timetree. **A**. Phylogeny showing selective optima with unique shifts per life phase marked as blue circle, and shared shifts between selective optima in adult and tadpole phenotypes marked with red circles. **B**. Selective optima for tadpoles and adults plotted against first two MDS variables for morphology. Colors in adult and tadpole trees are not comparable. **C**. Shifts among selective phenotypic optima are occurring more often than expected by random on the same nodes. ** denotes significant result with p<0.01.

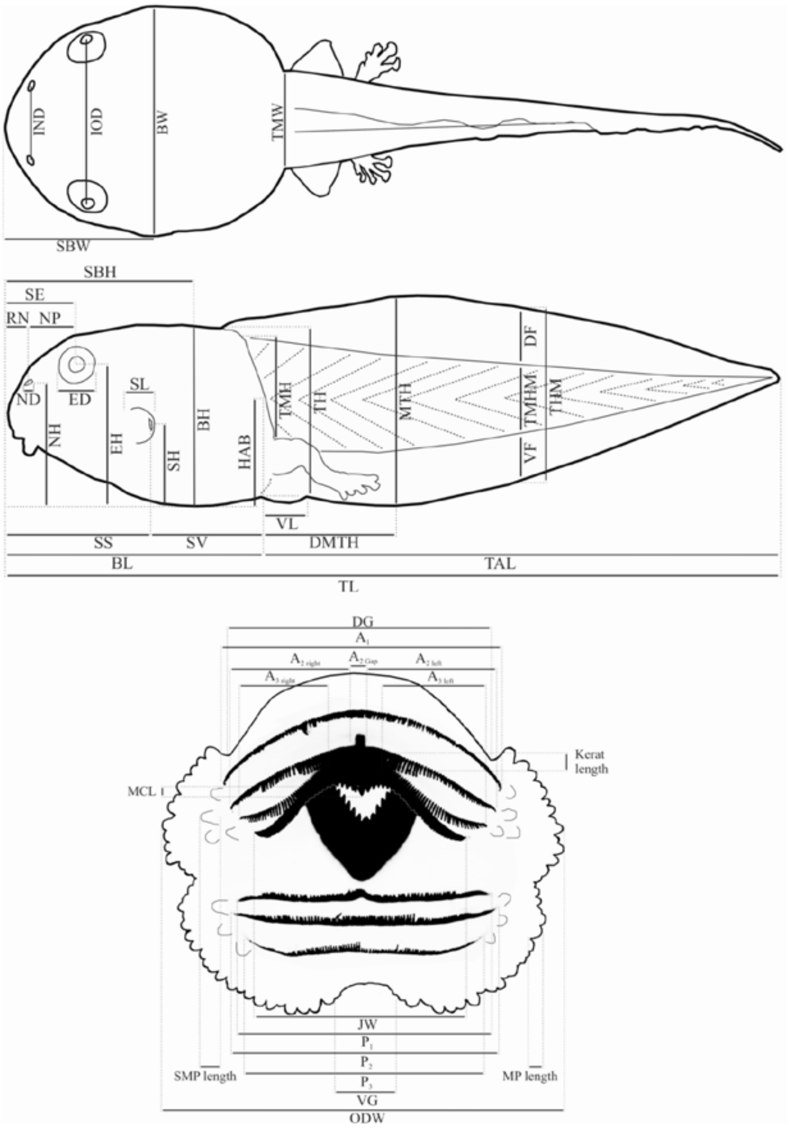

Supplementary Figure 8. Schematic drawing of a tadpole in dorsal and lateral view, and of tadpole mouthparts, showing abbreviations of measurements and counts as used for scoring larval character states (Supplementary Table 5).

Supplementary References

1. AmphibiaWeb. Information on amphibian biology and conservation.[web application]. Berkeley, California: AmphibiaWeb (2017).
2. Vieites, D.R., Wollenberg, K.C., Andreone, F., Köhler, J., Glaw, F., Vences, M. Vast underestimation of Madagascar's biodiversity evidenced by an integrative amphibian inventory. *Proc. Natl. Acad. Sci. U.S.A.* **106**, 8267–8272 (2009).
3. Perl, R.G.B., Nagy, Z.T., Sonet, G., Glaw, F., Wollenberg, K.C., Vences, M. DNA barcoding Madagascar's amphibian fauna. *Amphibia-Reptilia* **35**, 197–206 (2014).
4. Padial, J.M., Miralles, A., De la Riva, I., Vences, M. The integrative future of taxonomy. *Front. Zool.* **7**, 16 (2010).
5. Encyclopedia of Life, available from "http://www.eol.org/pages/332846". "*Boophis lichenoides* Vallan, Glaw, Andreone and Cadle, 1998". Accessed 26 Aug 2011.
6. Andreone, F. Aprea, G., Vences, M., Odierna, G. A new frog of the genus *Mantidactylus* from the rainforests of north-eastern Madagascar, and its karyological affinities. *Amphibia-Reptilia* **24**, 285–303 (2003).
7. Andreone, F., Nussbaum, R.A. A revision of *Mantidactylus microtis* and *M. microtympanum*, and a comparison with other large Madagascan stream frogs (Anura: Mantellidae: Mantellinae). *Zootaxa* **1105**, 49–68 (2006).
8. Andreone, F., Rosa, G.M., Noël, J., Crottini, A., Vences, M., Raxworthy, C.J. Living within fallen palm leaves: the discovery of an unknown *Blommersia* (Mantellidae: Anura) reveals a new reproductive strategy in the amphibians of Madagascar. *Naturwissenschaften* **97**, 525–543 (2010).
9. Crottini, A., Glaw, F., Casiraghi, M., Jenkins, R.K.B., Mercurio, V., Randrianantoandro, J.C., Randrianirina, J.E., Andreone, F. A new *Gephyromantis* (*Phylacomantis*) frog species from the pinnacle karst of Bemaraha, western Madagascar. *ZooKeys* **81**, 51–71 (2011).
10. Glaw, F, Vences, M. A Field Guide to the Amphibians and Reptiles of Madagascar. Third Edition. Cologne, Germany (2007).
11. Duellman, W.E., Lehr, E. Terrestrial-breeding frogs (Strabomantidae) in Peru. Natur und Tier. Berlin, Germany (2009).
12. Glaw, F., Vences, M. Resurrection and redescription of *Mantidactylus tricinctus* from Eastern Madagascar. *J. Herpetol.* **33**, 639–647 (1999).
13. Glaw, F., Vences, M., Gossmann, V. A new species of *Mantidactylus* (subgenus *Guibemantis*) from Madagascar, with a comparative survey of internal femoral gland structure in the genus (Amphibia: Ranidae: Mantellinae). *J. Nat. Hist.* **34**, 1135–1154 (2000).
14. Glaw, F., Vences, M. Two new sibling species of *Mantidactylus cornutus* from Madagascar (Amphibia, Anura, Ranidae). *Spixiana* **24,** 177–190 (2001).
15. Glaw, F., Vences, M., Andreone, F., Vallan, D. Revision of the *Boophis majori* group (Amphibia: Mantellidae) from Madagascar, with descriptions of five new species. *Zool. J. Linn. Soc.* **133**, 495–529 (2001).
16. Glaw, F., Hoegg, S., Vences, M. Discovery of a new basal relict lineage of Madagascar frogs and its implications for mantellid evolution. *Zootaxa* **1334**, 27–43 (2006).
17. Glaw, F., Vences, M. A new sibling species of the anuran subgenus *Blommersia* from Madagascar (Amphibia: Mantellidae: *Mantidactylus*) and its molecular phylogenetic relationships. *Herpetol. J.* **12**, 11–20 (2002).
18. Glaw, F., Vences, M. A new cryptic treefrog species of the *Boophis luteus* group from Madagascar: bioacoustic and genetic evidence. *Spixiana* **25**, 173–181 (2002).
19. Glaw, F., Vences, M. A new cryptic frog species of the *Mantidactylus boulengeri* group with a divergent vocal sac structure. *Amphibia-Reptilia* **23**, 293–304 (2002).
20. Glaw, F., Vences, M. A new species of *Mantidactylus* (Anura: Mantellidae) from Andasibe in Eastern Madagascar. *J. Herpetol.* **36**, 372–378 (2002).
21. Glaw, F., Vences, M. A preliminary review of cryptic diversity in frogs of the subgenus *Ochthomantis* based on mtDNA sequence data and morphology (Anura, Mantellidae, *Mantidactylus*). *Spixiana* **27**, 83–81 (2004).
22. Glaw, F, Vences, M. Phylogeny and genus. level classification of mantellid frogs (Amphibia, Anura). *Org. Divers. Evol.* **6**, 236–253 (2006).
23. Glaw, F., Köhler, J., Vences, M. New species of *Gephyromantis* from Marojejy National Park, Northeast Madagascar. *J. Herpetol.* **45**, 155–160 (2011).

24. Bora, P., Ramilijaona, O., Raminosoa, N., Vences, M. A new species of *Mantidactylus* (subgenus *Chonomantis*) from Ranomafana National Park, eastern Madagascar (Amphibia, Anura, Mantellidae). *Zootaxa* **2772**, 52–60 (2011).
25. Pabijan, M., Gehring, P.S., Köhler, J., Glaw, F., Vences, M. A new microendemic frog species of the genus *Blommersia* (Anura: Mantellidae) from the east coast of Madagascar. *Zootaxa* **2978**, 34–50 (2011).
26. Vallan, D., Glaw, F., Vences, M. Two new species of the *Boophis mandraka* complex (Anura, Mantellidae) from the Andasibe region in eastern Madagascar. *Amphibia-Reptilia* **24**, 305–319 (2004).
27. Vences, M., Glaw, F., Andreone, F. Description of two new frogs of the genus *Mantidactylus* from Madagascar, with notes on *Mantidactylus klemmeri* (Guibé, 1974) and *Mantidactylus webbi* (Grandison, 1953) (Amphibian, Ranidae, Mantellinae). *Alytes* **14**, 130–146 (1997).
28. Vences, M., Andreone, F., Glos, J., Glaw, F. Molecular and bioacoustic differentiation of *Boophis occidentalis* with description of a new treefrog from north-western Madagascar. *Zootaxa* **2544**, 54–68 (2010).
29. Vences, M., Glaw, F. Two new treefrogs of the *Boophis rappiodes* group from eastern Madagascar (Amphibia Mantellidae). *Tropical Zool.* **15**, 141–163 (2002).
30. Vences, M., Glaw, F. Revision of the subgenus *Chonomantis* (Anura: Mantellidae: *Mantidactylus*) from Madagascar, with description of two new species. *J. Nat. Hist.* **38**, 77–118 (2004).
31. Vences, M., Glaw, F. A new cryptic frog of the genus *Boophis* from the north-western rainforests of Madagascar. *Afr. J. Herpetol.* **54**, 7–84 (2005).
32. Vences, M., Andreone, F., Vieites, D.R. New treefrog of the genus *Boophis* Tschudi 1838 from the northwestern rainforests of Madagascar. *Trop. Zool.* **18**, 237–249 (2005).
33. Vences, M., Glaw, F. A new species of *Mantidactylus* from the east coast of Madagascar and its molecular phylogenetic relationships within the subgenus *Guibemantis*. *Herpetol. J.* **15**, 37–44 (2005)
34. Wollenberg, K.C., Andreone, F., Glaw, F., Vences M. Pretty in pink: A new treefrog species of the genus *Boophis* from North-Eastern Madagascar. *Zootaxa* **1684**, 58–68 (2008).
35. Amat, F., Wollenberg, K.C. and Vences, M. Correlates of eye colour and pattern in mantellid frogs. *Salamandra* **49**, 7–17 (2013).
36. Wollenberg, K.C., Vieites, D.R., Glaw, F., Vences, M. Speciation in little: the role of range and body size in the diversification of Malagasy mantellid frogs. *BMC Evol. Biol.* **11**, 217 (2011).
37. Grosjean, S., Strauß, A., Glos, J., Randrianiaina, R.D., Ohler, A., Vences, M. Morphological and ecological uniformity in the funnel-mouthed tadpoles of Malagasy litter frogs, subgenus *Chonomantis*. *Zool. J. Linn. Soc.* **162**, 149-183 (2011).
38. Jovanovic, O., Glos, J., Glaw, F., Randrianiaina, R., Vences, M. Comparative larval morphology of *Mantella*. *Zootaxa* **2124**, 21–37 (2009).
39. Knoll, A., Köhler, J., Glaw, F., Teschke, M., Vences, M. Larval morphology in four species of Madagascan frogs of the subgenus *Brygoomantis* (Mantellidae: *Mantidactylus*). *Zootaxa* **1616**, 49–59 (2007).
40. Raharivololoniaina, L., Grosjean, S., Raminosoa, N., Glaw, F., Vences, M. Molecular identification, description and phylogenetic implications of the tadpoles of 11 species of Malagasy treefrogs, genus *Boophis*. *J. Nat. Hist.* **40**, 1449–1480 (2006).
41. Randrianiaina, R.D., Glaw, F., Thomas, M., Glos, J., Raminosoa, N., Vences, M. Descriptions of the tadpoles of two species of *Gephyromantis*, with a discussion of the phylogenetic origin of direct development in mantellid frogs. *Zootaxa* **1401**, 53–61 (2007).
42. Randrianiaina, R.D., Raharivololoniaina, L., Preuss, C., Strauß, A., Glaw, F., Teschke, M., Glos, J., Raminosoa, N., Vences, M. Descriptions of the tadpoles of seven species of Malagasy treefrogs, genus *Boophis*. *Zootaxa* **2021**, 23–41 (2009).
43. Randrianiaina, R.D., Strauß, A., Glos, J., Glaw, F., Vences, M. Diversity, external morphology and 'reverse taxonomy' in the specialized tadpoles of Malagasy river bank frogs of the subgenus *Ochthomantis* (genus *Mantidactylus*). *Contrib. Zool.* **80**, 17-65 (2011)
44. Randrianiaina, R.D., Wollenberg, K.C., Rasolonjatovo Hiobiarilanto, T., Strauß, A., Glos, J., Vences, M. Nidicolous tadpoles rather than direct development in Malagasy frogs of the genus *Gephyromantis*. *J. Nat. Hist.* **45**, 2871-2900 (2011).
45. Randrianiaina, R.D., Strauß, A., Glos, J., Vences, M. Diversity of strongly rheophilous tadpoles of Malagasy tree frogs, genus *Boophis* (Anura, Mantellidae), and identification of new candidate species via larval DNA sequence and morphology. *ZooKeys* **178**, 59-124 (2012).
46. Schmidt, H., Strauß, A., Glaw, F., Teschke, M., Vences, M. Description of tadpoles of five frog species in the subgenus *Brygoomantis* from Madagascar (Mantellidae: *Mantidactylus*). *Zootaxa* **1988**, 48–60 (2009).
47. Strauss, A. Reeve, E. Randrianiaina, R.D. Vences, M. Glos, J. The world's richest tadpole communities show functional redundancy and low functional diversity: ecological data on Madagascar's stream-dwelling amphibian larvae. *BMC Ecol.* **10**, 12 (2010).

48. Hansen, T.F., Pienaar, J., Orzack, S.H. A comparative method for studying adaptation to a randomly evolving environment. *Evolution* **62**, 1965–1977 (2008).
49. Butler, M.A., King, A.A. Phylogenetic comparative analysis: a modeling approach for adaptive evolution. *Amer. Nat.* **164**, 683–695 (2004).
50. Ingram, T., Mahler, D.L. SURFACE: detecting convergent evolution from comparative data by fitting Ornstein-Uhlenbeck models with stepwise Akaike Information Criterion. *Methods Ecol. Evol.* **4**, 416–425 (2013).
51. Pagel, M., Meade, A., Barker, D. Bayesian estimation of ancestral character states on phylogenies. *Syst. Biol.* **53**, 673–684 (2004).
52. Pagel, M. Detecting correlated evolution on phylogenies: A general method for the comparative analysis of discrete characters. *Proc. R. Soc. London B* 255, 37–45 (1994).
53. Barker, D., Pagel, M.. Predicting functional gene links from phylogenetic-statistical analyses of whole genomes. *PLoS Comput. Biol.* **1**, e3 (2005).
54. Strimmer, K. fdrtools: a versatile R package for estimating local and tail area-based false discovery rates. *Bioinformatics* **24**, 1461–1462 (2008).
